# Current reproductive effort shapes the response to infection in a passerine bird

**DOI:** 10.1111/1365-2656.70086

**Published:** 2025-06-27

**Authors:** Fredrik Andreasson, Andreas Nord, Arne Hegemann, Jan‐Åke Nilsson

**Affiliations:** ^1^ Department of Biology Lund University Lund Sweden

**Keywords:** hyperthermia, immune function, infection, life‐history theory, parental effort, terminal investment

## Abstract

1. When infected with a pathogen, a host may respond with an acute phase response to increase its chances of survival. Previous research shows that one fundamental component of the acute phase response, adjustments of body temperature, may differ depending on ambient temperature, social settings and energy availability.
2. However, we do not know much about how such a response is modulated during work‐intensive reproductive events.
3. Therefore, we subjected breeding female blue tits *Cyanistes caeruleus* that were raising experimentally enlarged broods or normal‐sized broods to a mimicked bacterial infection. We quantified their subsequent body temperature response and haptoglobin concentrations together with the effect on parental work rate and breeding performance.
4. Immune challenged females with enlarged broods initially showed hypothermia while immune challenged females with normal sized broods instead developed fever. Additionally, immune challenged females with normal broods had higher circulating haptoglobin levels compared to immune challenged females caring for enlarged broods. Thus, females that were ‘doubly’ challenged with both an enlarged brood and an immune challenge mounted a lesser immune response but still managed to sustain nestling growth comparable to nestlings from normal sized broods.
5. Hence, our results show that experimental manipulation of brood size shapes the acute phase response and the trade‐off between self‐maintenance and current reproduction.

## Graphical abstract

Our study fills a much‐needed gap and connects the body temperature response to infection with the framework of reproductive investment and trade‐offs between current reproduction and self‐maintenance. Thus, we think that our study highlights a general, unexplored connection between physiology and life‐history theory.

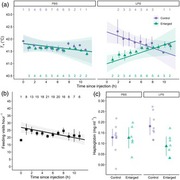

## 1 INTRODUCTION

Pathogens can reduce host fitness by causing direct physiological damage or by depletion of host resources (Connors & Nickol, 1991) but also indirectly via over‐activation of the immune response itself (reviewed in Medzhitov et al., 2012). The fitness consequences of such effects may vary between annual‐cycle stages and species but are often particularly critical during the reproductive season (Martin et al., 2008). To avoid infection or limit its detrimental effects, a well‐functioning immune defence system is needed. However, activation of an immune response can be costly and mandate reductions in, for example, parental effort (Bonneaud et al., 2003; Ilmonen et al., 2000; Råberg et al., 2000) with downstream consequences for the offspring (Bonneaud et al., 2003; Ilmonen et al., 2000). These shifts in parental time and energy budgets can be caused by sickness behaviours (Burness et al., 2010; Sköld‐Chiriac et al., 2014), as a protection against immunopathology (Hasselquist & Nilsson, 2012) and/or by production costs of the immune response (Hawley et al., 2012; Hegemann, Matson, Flinks, et al., 2013). Thus, for reproducing animals, the cost of an immune response will be an important factor when balancing future reproductive potential against the value of the current breeding attempt (Hasselquist & Nilsson, 2012; Sheldon & Verhulst, 1996).

The first reaction to infection, the acute phase response, is highly conserved in vertebrates and generally entails both physiological changes that increase energy demands and may manifest in fever (Kluger, 1991; Marais, Gugushe, et al., 2011; Marais et al., 2011a) and behavioural changes that alleviate energy costs (Hart, 1988; for avian examples, see Adelman, Bentley, et al., 2010; Adelman, Córdoba‐Córdoba, et al., 2010; Burness et al., 2010; Lennon et al., 2023; Owen‐Ashley et al., 2006; Sköld‐Chiriac et al., 2014). Fever is regulated similarly in mammals and birds with prostaglandins, nitric oxide and cytokines mediating avian febrile responses (Gray et al., 2005; Johnson et al., 1993; Marais et al., 2011b; Nomoto, 2003; reviewed by Gray et al., 2013) where, more specifically, Toll‐like receptors (Martin et al., 2014; Scalf et al., 2019) and interleukins IL‐6 (Grabbe et al., 2020; Lopes et al., 2012; Martin et al., 2014) and IL‐1β (Lopes et al., 2013; Scalf et al., 2019) are upregulated as a response to an endotoxin challenge. The beneficial aspects of fever are generally thought to be linked to reduced proliferation of pathogens and improved immune function (Kluger et al., 1996). Generally, a 1°C increase is thought to increase metabolic costs by 10% (Kluger & Rothenburg, 1979), but this increase could be higher in birds (Marais et al., 2011a). However, in addition to any direct energetic cost associated with a febrile response during the acute phase response, there are also indirect costs. Reduced food intake may cause nutritional constraints (Lochmiller & Deerenberg, 2000) and the response to infection will increase the levels of reactive oxygen species (ROS), thereby increasing levels of oxidative stress and damage (Armour et al., 2020; Baylor & Butler, 2019; Bryla et al., 2022; Casasole et al., 2016; Costantini & Dell'Omo, 2006; Hõrak et al., 2007; Stier et al., 2009; but see Cram et al., 2015) and the risk for immunopathology (Hasselquist & Nilsson, 2012; Råberg et al., 1998).

Thus, it is perhaps not surprising that the trade‐off between immune response and other life‐history components such as reproduction and self‐maintenance yields different, sometimes even contrasting, outcomes depending on context and species. For example, activity is not always reduced after an endotoxin challenge when the animal is challenged in a socially competitive situation (Lopes et al., 2012), while individuals of highly social species might reduce contact with conspecifics (Stockmaier et al., 2018) or even isolate themselves after an immune challenge (Moreno et al., 2021). Likewise, febrile or metabolic responses to infection, as well as the production of immune response regulators, may be attenuated or reversed in energetically demanding situations (Amaral‐Silva et al., 2022; Liu et al., 2012; but see Nord et al., 2013), in socially competitive settings (Vaziri et al., 2019) or when the animal is in an energy‐conserving mode (Nord et al., 2020; Romanovsky & Székely, 1998).

So far, reported body temperature responses during the acute phase response in birds show both diurnal variation (Nomoto, 1996) and size dependence. Generally, large (>50 g), precocial birds exhibit fever when challenged with bacterial endotoxin (Koutsos & Klasing, 2001; Leshchinsky & Klasing, 2001; Maloney & Gray, 1998; Marais et al., 2011a; Marais, Gugushe, et al., 2011; but see Nord et al., 2020). Small passerines (10–50 g) tend to get fever if the acute phase response is initiated in the evening or during the night (Adelman, Bentley, et al., 2010; Adelman, Córdoba‐Córdoba, et al., 2010; Coon et al., 2011; Hegemann et al., 2012; Nord et al., 2013; Sköld‐Chiriac et al., 2015) but show hypothermic responses during the day (Burness et al., 2010; King & Swanson, 2013; Owen‐Ashley et al., 2006; Sköld‐Chiriac et al., 2015; but see Adelman, Bentley, et al., 2010; Adelman, Córdoba‐Córdoba, et al., 2010; Tapper et al., 2022). Possible mechanisms for such diurnal hypothermic responses include downregulated expression of genes that regulate metabolism and upregulation of immune genes (Scalf et al., 2019). While several studies have investigated body temperature changes during an acute phase response in birds, no studies have addressed this response specifically during reproduction, when altricial species typically engage in intensive, time‐ and energy‐demanding parental care. Under such circumstances, the readiness to pay the cost of mounting an immune response appears to be workload dependent (reviewed in Knowles et al., 2009) and may not be detectable when individuals trade off investment in immune function with investment in reproductive effort (Bonneaud et al., 2003). Thus, it is likely that birds will modulate the strength (and maybe even direction) of the immune response with changes in workload, which may result in hypo‐ or hyperthermia, respectively.

Another element of the acute phase response is the production of acute phase proteins. These proteins inhibit microbial growth and facilitate restoration of homeostasis after infection, inflammation or trauma (Murata et al., 2004). One of these proteins, haptoglobin (or its functional equivalent, i.e. PIT54; see Wicher & Fries, 2006), is produced and released by the liver as a response to cytokines released during infection (Baumann & Gauldie, 1994; Cray et al., 2009; Quaye, 2008) and by binding free haemoglobin from damaged erythrocytes, it prevents oxidative damage and removes iron needed for bacterial growth (Gutteridge, 1987; Langlois & Delanghe, 1996). In birds, haptoglobin concentration has been shown to increase after an immune challenge (Armour et al., 2020; Buehler et al., 2009; George et al., 2017; Matson et al., 2012; Millet et al., 2007; van de Crommenacker et al., 2010; but see Coon et al., 2011; Hegemann, Matson, Versteegh, et al., 2013; Nord et al., 2020; Schultz et al., 2017) and can therefore provide additional information on the magnitude of the immune response, and importantly, it can do so on a longer time‐scale compared to the acute body temperature response (George et al., 2017).

To test whether increased reproductive effort affected the response to infection, we enlarged blue tit *Cyanistes caeruleus* broods (c.f. García‐Navas & Sanz, 2010; Nur, 1984), subjected the breeding females to a mimicked bacterial infection (using lipopolysaccharide; henceforth LPS) and measured subsequent feeding frequency, body temperature and haptoglobin concentration. Together with measurements of nestling biometry at 14 days of age, this allowed us to test if the response to infection also affected parental reproductive investment and thereby the trade‐off between self‐maintenance and current reproduction.

## 2 METHODS

### 2.1 Study population

Fieldwork was performed during the breeding season (May–June) in 2018, in our nest‐box population centred around Lake Krankesjön (55°42′ N, 13°28′ E), ca. 20 km outside of Lund in the south of Sweden (see Källander et al., 2017 for a more detailed description of the study area). The area contained ca. 600 nest‐boxes at the time of the study. Blue tits in our population usually lay the first eggs by mid to late April. When the clutch is completed, the eggs are incubated for at least 12 days by the female after which nestlings are fed by both parents until they fledge some 3 weeks after hatching. Early in the season, we checked nests at least weekly to determine the start of egg laying. Starting at incubation day 12, we checked nests daily for hatching (day of hatching = nestling day 0). Ambient temperature, *T*_a_, can influence body temperature (Tapper et al., 2020) and foraging activity (du Plessis et al., 2012) and was measured in 30 min intervals with an iButton (DS1922‐L, Sunnyvale, CA, USA; accuracy ±0.5°C, resolution ±0.0625°C) placed in the shade, 1.5 m above ground, in the centre of the study area. Experimental protocols were approved by Malmö/Lund Animal Ethics Committee (permit no. 5.8.18‐04705/2018). Catching and ringing of birds was performed under the permission of the Swedish Ringing Centre (licence no. 475).

### 2.2 Nestling day 6—Brood size manipulations

When nestlings were 6 days old, nest‐boxes with 9–13 nestlings were randomly assigned to one of two brood categories: experimentally enlarged or normal‐sized control broods (henceforth ‘enlarged’ and ‘control’, respectively). Nestlings in both brood categories were ringed and weighed, the only difference being that enlarged broods also received five nestlings that were transferred from a donor nest of the same age (donor nests were not included in the study). Due to constraints on brood availability, nestlings were not moved between control nests. In total, 19 nests were assigned to the enlarged category (mean brood size after manipulation ± SD: 15.4 ± 0.8 nestlings) and 20 nests were assigned to the control category (mean brood size ± SD: 10.4 ± 1.2 nestlings). For comparison, mean (±SD) clutch size in the following 3 years (2019–2021) was 10.4 (±1.7) and our manipulated, enlarged broods (14–17 nestlings) were within the natural range of clutch sizes in the population (≤18). Mean body mass of nestlings after the brood size manipulation on nestling day 6 did not differ between the four experimental groups (ANOVA: *F*_3,35_ = 0.5, *p* = 0.70, see Table 1 for sample and brood sizes and additional group descriptives). Nestlings that were transferred from donor nests to enlarged nests had lower body mass compared to nestlings in the recipient nest‐box on nestling day 14 in one of the experimental categories (see Figure S1; Table S1). Therefore, we included the effect of transfer in our models of nestling biometry (see Section 2.6). Our main aim was to investigate responses to infection during hard work, and thus, we did not include a reduced brood category. On nestling day 10, broods from both brood categories (enlarged and control) were assigned to two treatments where the female parent was either immune challenged or sham injected, creating a 2 × 2 factorial design.

**TABLE 1:** Summary statistics of key variables in the four experimental groups.

Key variables	PBS‐C	PBS‐E	LPS‐C	LPS‐E
For all nests at the start of the experiment				
Total number of nests	10	8	10	11
Mean brood size (±SD) before brood size manipulation	10.00 ± 1.05	9.75 ± 0.71	10.70 ± 1.25	10.82 ± 0.60
Brood size range after brood size manipulation	9–12	14–16	9–13	15–17
Mean nestling body mass (g ± SD) after brood size manipulation on nestling day 6	6.55 ± 0.70	6.34 ± 0.30	6.58 ± 0.82	6.34 ± 0.43
For nests included in analyses of *T*_s_ and feeding frequency at nestling day 10				
Hatching (Julian day, ±SD)	136.5 ± 4.1	136.2 ± 4.4	136.5 ± 3.9	137.5 ± 2.8
Sample size	8	5	6	6
For nests at nestling day 14				
Number of nests where female was feeding at day 14	9	6	8	8
Number of nests where both parents were feeding on day 14 (included in day 14 analyses)	9	6	8	7
Brood size (±SD) on day 14	9.00 ± 1.66	13.67 ± 1.51	10.50 ± 1.20	15.14 ± 1.57
Median number of nestlings lost between day 6 and day 14	0	1	0	0
Hatching (Julian day, ±SD)	137.1 ± 4.2	135.5 ± 3.8	136.8 ± 4.3	136.9 ± 3.2

Abbreviations: LPS‐C, un‐manipulated, normal‐sized broods raised by a female receiving a mimicked bacterial infection triggered by a lipopolysaccharide (LPS) injection; LPS‐E, experimentally enlarged broods raised by a female receiving a mimicked bacterial infection; PBS‐C, un‐manipulated, normal‐sized broods raised by a female blue tit receiving a sham injection with PBS; PBS‐E, experimentally enlarged broods raised by a female receiving a sham‐injection with PBS.

### 2.3 Nestling day 7—PIT‐tagging of adults

When nestlings were 7 days old, both parents were caught in the nest‐box while feeding nestlings, ringed, weighed (±0.1 g) and measured (tarsus ±0.1 mm, wing ±0.5 mm). Parents were aged based on plumage characteristics (Svensson, 1992) and sex was determined based on the presence/absence of a brood patch. To measure body temperature in females, we implanted a temperature‐sensitive passive integrated transponder (PIT‐tag, BioTherm13, Biomark, Boise, ID, USA; height: 13.0 mm; diameter: 2.1 mm) subcutaneously in the neck under aseptic conditions and sealed the incision with cyanoacrylate (Loctite Power Easy Gel, Henkel AG & Company, Düsseldorf, Germany). This tagging method does not affect survival or body mass in another small passerine, the black‐capped chickadee *Poecile atricapillus* (Farr et al., 2021). All females were implanted, and thus, any energy costs from healing the incision or recovering from the implantation did not differ between the experimental groups. Subcutaneous implants have previously been shown to correlate well with core body temperature in other small passerines (Andreasson et al., 2023; Nord et al., 2013; Sköld‐Chiriac et al., 2015). In a cross‐sectional study on great tits *Parus major*, Andreasson et al. (2023) found that body temperature measured by subcutaneous implants did not differ from cloacal temperature or from body temperature recorded by intraperitoneal implants across a wide range of ambient temperatures. On a within‐individual level, subcutaneous implants have been shown to record temperatures 0.5–1.5°C below cloacal temperature in great tits (Nord et al., 2013) and zebra finches *Taeniopygia guttata* (Sköld‐Chiriac et al., 2015). Males were fitted with the same type of PIT‐tag taped to two plastic rings on the tarsus to monitor feeding frequency.

### 2.4 Nestling day 10—Immune challenge in adults, measurements of female body temperature and parental feeding frequency

When nestlings were 10 days old, females assigned to the immune challenge group were injected subcutaneously in the abdominal area with LPS (*E. coli* 055:B5 LPS [Sigma‐Aldrich no. L2880], 1 μg g^−1^ bird body mass, dissolved in 50 μL phosphate‐buffered saline [PBS]). Injection with a bacterial lipopolysaccharide mimics a bacterial infection and initiates an acute phase response, including metabolic responses, in birds (Marais, Gugushe, et al., 2011; Marais et al., 2011a; Nord et al., 2013; Sköld‐Chiriac et al., 2014, 2015). The sham injected females were injected with PBS (50 μL). These experimental groups are from here on referred to as PBS‐C (PBS injection and control brood category), PBS‐E (PBS injection and enlarged brood category), LPS‐C (LPS injection and control brood category) and LPS‐E (LPS injection and enlarged brood category). Initially, assignment to the immune challenge categories was random, but as the breeding season progressed, broods were assigned non‐randomly to account for nests that were lost due to predation and abandonment. Immediately after the injection, we placed a PIT‐tag reader (Biomark HPR Plus, Biomark, Boise, ID, USA) connected to a circular antenna under each nest‐box to record subcutaneous body temperature, *T*_s_, of the female and feeding frequency of both parents during approximately 24 h (mean ± SD duration of recording: 24.5 ± 2.4 h, range: 19.3–30.7 h). The readers recorded individual PIT‐tags with a 60 s refractory period (i.e. after an individual had been recorded it could not be recorded again within 60 s). With minor modifications, we followed the technique described by Andreasson et al. (2020) to filter out possible double readings (see Supporting Information for a detailed description).

### 2.5 Nestling day 14—Biometric measurements and blood sampling

When nestlings were 14 days old, we weighed and measured tarsus and wing length of nestlings and recaptured parents. Four females were recaptured and sampled after day 14 (day 15: *n* = 2 [LPS‐C and LPS‐E], day 16: *n* = 1 [PBS‐E], day 17: *n* = 1 [LPS‐C]) but were still included in the analyses. Two nestlings from one nest‐box (LPS‐C) were identified as marsh tits (*Poecile palustris*) at day 14 and were excluded from analyses of nestling biometry. When parents were captured, we also collected a blood sample (jugular vein, 100 μL) from them. The blood samples were stored in heparinized Eppendorf tubes and directly chilled on ice. Within 5 h of blood sampling, samples were centrifuged for 10 min at 4000 rpm, plasma was separated from cells and samples were subsequently frozen (−50°C) until analysis of haptoglobin concentration 8–10 weeks later. Haptoglobin concentrations were measured with a colorimetric kit (TP801; Tri‐Delta Diagnostics, Maynooth, Ireland). In addition to the standard protocol issued by the kit manufacturer, we also measured absorbance at 405 and 450 nm to control for differences in plasma redness (Matson et al., 2012; Wemer et al., 2021).

### 2.6 Data analyses and sample sizes

**FIGURE 1:**
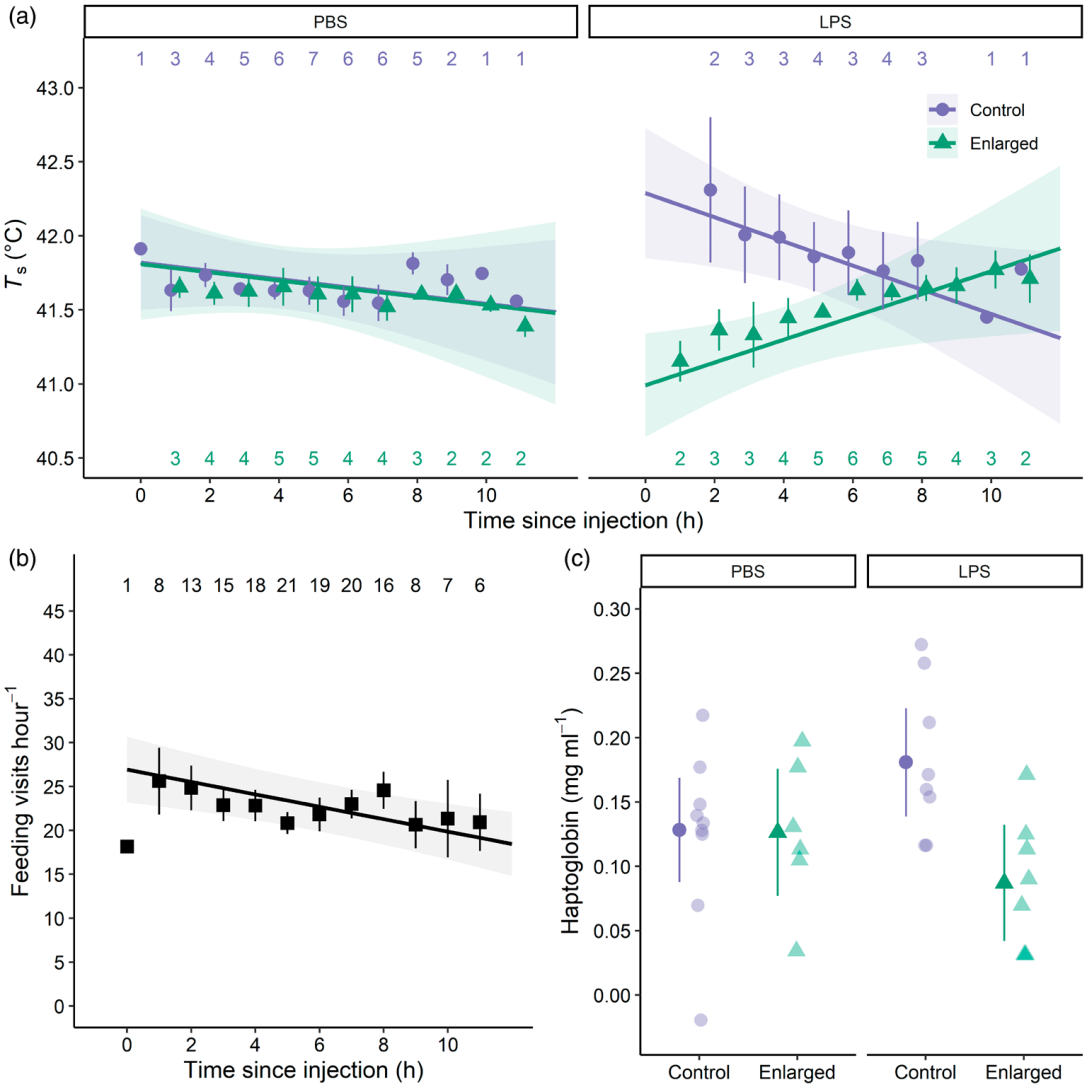
(a) Subcutaneous body temperature, *T*_s_, (b) feeding frequency and (c) haptoglobin concentration in blue tit females with enlarged or control brood sizes, after being injected with a bacterial endotoxin (lipopolysaccharide, LPS) or a saline solution (phosphate‐buffered saline, PBS). *T*_s_ and feeding frequency were recorded on the day of injection while haptoglobin was measured 4 days later. Numbers represent hourly sample sizes. In (a) and (b), model regressions together with their 95% CI are shown together with raw data points (±SE). In (c), model estimates (±95% CI) are indicated by solid symbols and individual raw data points are depicted with semi‐transparent symbols.

The strongest body temperature response to mimicked infections in passerines usually occurs after 2–4 h (Owen‐Ashley et al., 2006 [white‐crowned sparrow *Zonotrichia leucophrys gambelii*]; Burness et al., 2010 [zebra finch]; King & Swanson, 2013 [house sparrow *Passer domesticus*]; Sköld‐Chiriac et al., 2015 [zebra finch]) but can extend for ≥6 h during the day (King & Swanson, 2013; Sköld‐Chiriac et al., 2015). Most injections were made before noon (mean injection time ± SD: 11:03 AM ± 118 min, range: 8:10 AM to 4:00 PM) and we analysed *T*_s_ and female feeding frequency during the remainder of the day until 8 PM (local time: GMT + 2). We chose to exclude measurements after 8 PM (sunset ranged from 9:20 to 9:39 PM over the experimental period) from the analysis to avoid including drops in *T*_s_ and feeding frequency during the period before going to roost, which would introduce variation unrelated to the experimental effect (see Figure S2). Of the 39 females included at the start of the experiment, some did not return to feed until the next day and some did not register any feeding events during the whole time the reader was deployed (see sample sizes for *T*_s_ and feeding frequency measurements in Table 1 and for a detailed table with nest‐box specific information, see Table S2). However, there was no difference between the brood size and immune challenge categories in the number of females that did not return on the day of injection (Fisher's exact test, *p* = 1, Table S2). For the females that did return during the day of injection, there was no difference between the brood size and immune challenge categories in the time it took for females to resume feeding after injection (see Figure S3). Hourly sample sizes are given in Figure 1.

Nests that were depredated (*n* = 1) or where one (*n* = 4) or both (*n* = 4) parents permanently abandoned the brood after the injection on day 10 were removed from the analysis of female immune function and body mass and nestling biometry (see Table 1; Table S2). Thus, the final sample size for those analyses was 30 nests with no difference in the permanent abandonment rate and/or depredation between the four categories (Fisher's exact test, *p* = 1).

To correct for interobserver variability (nestling biometrics were recorded by two observers, FA and AN), measurements of nestling tarsus and wing length were calibrated based on joint measurements of 58 adult individuals (see Figure S4).

All statistical analyses were done using R v.4.5.0 (http://www.R‐project.org/). We analysed feeding frequency (feeding visits h^−1^) and *T*_s_ using a linear mixed model (lmer function in lme4; Bates et al., 2015) with feeding frequency and *T*_s_ as dependent variables, brood size and immune challenge category as categorical fixed factors and time since injection (in hours), ambient temperature (*T*_a_), hatching date (Julian day) and time of injection (in hours, mean centred) as covariates. For models of feeding frequency, hour since injection and corresponding mean hourly *T*_a_ were used, while the *T*_a_—measurement closest in time to any given *T*_s_—reading was used for the models of *T*_s_ together with the time since injection (resolution of 1 s). For both models of *T*_s_ and feeding frequency, nest‐box (i.e. female ID) was included as a random factor and we compared a full model with both a random intercept and random slope (over time) to a full model with random intercept only, using AICc (Burnham & Anderson, 2002), to determine random structure. For the *T*_s_—model, the random structure with both intercept and slope was preferred (AICc = 862.1 vs. 1046.1 for intercept only) while for feeding frequency, the intercept only structure performed slightly better (AICc = 986.8 vs. 988.0 for both intercept and slope).

Because we wanted to evaluate the response to an experimental immune challenge over time since injection, we included the three‐way interaction ‘brood size category × immune challenge category × time’ and all underlying two‐way interactions. When females resumed feeding after the immune challenge the number of feeding visits during the first hour upon returning was extrapolated to a full hour unless females fed less than 15 min of the hour in which case that hour was excluded in its entirety. For example, if a female resumed feeding during the second hour after the challenge but only fed during the last 20 min of that hour the number of feeding visits for that hour was calculated as the number of visits under 20 min × 3. This was also done for the last hour of feeding measurements.

Since males may compensate for reduced female effort (Harrison et al., 2009) after injection, we analysed mean male feeding frequency from reader deployment to 8 PM using a two‐way ANCOVA, including the interaction between brood size and immune challenge categories with hatching date (Julian day) and time of injection as covariates. We modelled female immune function (haptoglobin) at day 14 in the same way (but without time of injection as a covariate) and analysed female body mass on nestling day 14 (as energetic stress during breeding can be reflected in mass loss, see Merilä & Wiggins, 1997) with a similar model, but here we also included body mass at day 10 as a covariate to control for initial differences in body mass and to avoid regression to the mean (see Clifton & Clifton, 2019). Female body mass at day 10 did not differ between the four groups (mean body mass [g] ± SD: PBS‐C 11.49 ± 0.36, PBS‐E 11.28 ± 0.25, LPS‐C 11.36 ± 0.49, LPS‐E 11.31 ± 0.38; ANOVA: *F*_3,26_ = 0.4, *p* = 0.73). For the model on haptoglobin, we also added absorbance at 405 nm as a covariate to account for differences in plasma redness after first comparing which of the two different wavelength absorbances (405 and 450 nm) that provided the best model fit (AICc 405 nm = −73.7, AICc 450 nm = −71.0).

Nestling biometry (body mass, tarsus and wing length) on day 14 was analysed in three separate linear mixed models with brood size and immune challenge category (and their interaction) as fixed factors, body mass at day 6 and hatching date (to account for a potential, general seasonal variation in nestling size) as covariates. Non‐independence between nestlings was accounted for by including nest‐box ID as a random intercept and the effect of moving nestlings was accounted for by the inclusion of nest‐box origin as a second random intercept.

All mixed models were fitted using restricted maximum likelihood, and degrees of freedom and p‐values were calculated using the Satterthwaite approximation using type III sums of squares (lmerTest, Kuznetsova et al., 2017). Estimates in the text and in Table 2 are estimated marginal means/trends and their 95% confidence intervals (CI) (emmeans, Lenth, 2024). Pairwise comparisons were adjusted for multiple comparisons using the *mvt* adjustment (emmeans, Lenth, 2024).

**TABLE 2:** Estimates, test statistics and *p*‐values for models on female subcutaneous body temperature (*T*_s_), feeding frequency and haptoglobin concentrations as well as nestling biometry in blue tits.

Variable	Estimate (95% CI)	df	*F*/*χ* ^2^	*p*
*T*_s_ (°C)				
Immune challenge		1, 16.2	1.0	0.33
Brood size category		1, 16.6	13.6	**0.002**
Time		1, 18.1	0.9	0.36
Immune challenge × Brood size category		1, 16.3	13.4	**0.002**
Immune challenge × Time		1, 17.6	0.6	0.44
Brood size category × Time		1, 17.6	6.2	**0.023**
Immune challenge × Brood size category × Time		1, 17.6	6.2	**0.023**
Slope				
PBS—Control	−0.028 (−0.086–0.030)			
PBS—Enlarged	−0.028 (−0.100–0.045)			
LPS—Control	−0.082 (−0.154 to −0.010)			
LPS—Enlarged	0.077 (0.011–0.143)			
*T*_a_	0.034 (0.023–0.045)	1, 2183.3	34.6	**<0.0001**
Hatching (Julian) date	0.018 (−0.012–0.049)	1, 17.4	1.6	0.23
Time of injection	−0.028 (−0.092–0.037)	1, 17.0	0.8	0.38
Nest‐box (random intercept + slope)		3	1265	**<0.0001**
Feeding frequency (feeding visits h^−1^)				
Immune challenge		1, 40.3	0.0	0.83
Brood size category		1, 41.6	1.1	0.31
Time	−0.71 (−1.12 to −0.29)	1, 136.3	11.4	**<0.001**
Immune challenge × Brood size category		1, 39.9	0.0	0.86
Immune challenge × Time		1, 140.2	0.5	0.49
Brood size category × Time		1, 139.6	1.5	0.23
Immune challenge × Brood size category × Time		1, 139.5	0.2	0.64
Slope				
PBS—Control	−0.41 (−1.09–0.27)			
PBS—Enlarged	−0.73 (−1.39 to −0.07)			
LPS—Control	−0.50 (−1.67–0.67)			
LPS—Enlarged	−1.20 (−1.84 to −0.56)			
*T*_a_	−0.65 (−1.28 to −0.01)	1, 137.6	4.1	**0.045**
Hatching (Julian) date	−0.26 (−1.07–0.54)	1, 17.2	0.5	0.50
Time of injection	−1.47 (−3.09–0.15)	1, 16.3	3.7	*0.073*
Nest‐box (random intercept)		1	42.9	**<0.0001**
Haptoglobin (mg mL^−1^)				
Immune challenge		1, 24	0.1	0.76
Brood size category		1, 24	4.9	**0.037**
Immune challenge × Brood size category		1, 24	4.5	**0.044**
PBS—Control	0.13 (0.09–0.17)			
PBS—Enlarged	0.13 (0.08–0.18)			
LPS—Control	0.18 (0.14–0.22)			
LPS—Enlarged	0.09 (0.04–0.13)			
Hatching (Julian) date	0.002 (−0.004–0.008)	1, 24	0.6	0.45
Absorbance at 405 nm	−0.28 (−0.65–0.09)	1, 24	2.4	0.13
Nestling wing length day 14 (mm)				
Immune challenge		1, 25.3	4.1	*0.053*
Brood size category		1, 25.6	1.4	0.24
Immune challenge × Brood size category		1, 25.2	6.4	**0.018**
PBS—Control	42.5 (41.1–43.9)			
PBS—Enlarged	40.0 (38.6–41.4)			
LPS—Control	42.2 (40.7–43.6)			
LPS—Enlarged	43.1 (41.8–44.4)			
Hatching (Julian) date	−0.04 (−0.23–0.15)	1, 25.6	0.1	0.70
Body mass day 6	2.17 (2.00–2.35)	1, 298.2	587.9	**<0.0001**
Nest‐box (random intercept)		1	0.9	0.34
Nest‐box origin (random intercept)		1	37.0	**<0.0001**
Nestling tarsus length day 14 (mm)				
Immune challenge		1, 21.6	0.7	0.40
Brood size		1, 22.1	0.8	0.37
Immune challenge × Brood size category		1, 21.5	3.5	*0.074*
PBS—Control	18.74 (18.43–19.05)			
PBS—Enlarged	18.33 (18.02–18.63)			
LPS—Control	18.59 (18.26–18.92)			
LPS—Enlarged	18.73 (18.45–19.01)			
Hatching (Julian) date	−0.03 (−0.07–0.02)	1, 21.8	1.8	0.20
Body mass day 6	0.27 (0.20–0.33)	1, 316.2	64.7	**<0.0001**
Nest‐box (random intercept)		1	0.4	0.52
Nest‐box origin (random intercept)		1	12.9	**<0.001**
Nestling body mass day 14 (g)				
Immune challenge		1, 24.1	1.9	0.18
Brood size		1, 24.4	1.6	0.22
Immune challenge × Brood size category		1, 24.1	2.0	0.17
PBS—Control	11.09 (10.47–11.71)			
PBS—Enlarged	10.26 (9.58–10.93)			
LPS—Control	11.07 (10.42–11.72)			
LPS—Enlarged	11.12 (10.50–11.75)			
Hatching (Julian) date	−0.13 (−0.21 to −0.04)	1, 25.3	8.7	**0.007**
Body mass day 6	0.52 (0.44–0.61)	1, 303.5	144.9	**<0.0001**
Nest‐box (random intercept)		1	1.8	0.18
Nest‐box origin (random intercept)		1	20.4	**<0.0001**

*Note*: *T*_s_ and feeding frequency were measured when nestlings were 10 days old after females with enlarged or normal‐sized broods were injected with a bacterial endotoxin (LPS) or phosphate‐buffered saline (PBS). Biometric measurements of nestlings were taken when nestlings were 14 days old, together with female haptoglobin concentrations. Significance values where *p* < 0.05 are given in bold font, and values where 0.1 > *p* > 0.05 are given in italics. Estimates are estimated marginal means and slopes with their 95% confidence intervals (CI).

## 3 RESULTS

### 3.1 Body temperature, feeding frequency, body mass and immune function

The immune challenge had a brood size category‐specific effect on *T*_s_ over the day of injection (brood size category × immune challenge category × time, *p* = 0.023; Table 2; Figure 1a). *T*_s_ over the day did not differ between PBS‐C (−0.028 [−0.086–0.030]°C h^−1^) and PBS‐E (−0.028 [−0.100–0.045]°C h^−1^, pairwise comparison: *p* = 1) females. In contrast, LPS‐E females had a lower *T*_s_ when they resumed feeding, but *T*_s_ then increased over time (0.077 [0.011–0.143]°C h^−1^) while LPS‐C females instead showed a higher *T*_s_ when they resumed feeding, and their body temperature subsequently decreased over time (−0.082 [−0.154 to −0.010]°C h^−1^, pairwise comparison: *p* = 0.010). This difference was not reflected in feeding frequency, which did not show a corresponding pattern (brood size category × immune challenge category × time, *p* = 0.64; Table 2; Figure 1b) and there was no brood size category‐specific (*p* = 0.31) or overall difference in feeding frequency between females challenged with LPS or injected with PBS (*p* = 0.83). Ambient temperature, *T*_a_, affected both *T*_s_ (*p* < 0.0001) and feeding frequency (*p* = 0.045) so that *T*_s_ increased with 0.034 (0.023–0.045)°C and females made 0.65 (1.28–0.01) fewer feeding visits h^−1^ for every °C increase in *T*_a_ (Table 2).

The effect of the immune challenge on female haptoglobin levels 4 days after experimental injection differed between females with broods of different sizes (brood size category × immune challenge category, *p* = 0.044; Table 2; Figure 1c), so that haptoglobin was higher in LPS‐C females (0.18 [0.14–0.22] mg mL^−1^) than in LPS‐E females (0.09 [0.04–0.13] mg mL^−1^, pairwise comparison: *p* = 0.015) while there was no such difference between PBS‐injected females with different brood sizes (pairwise comparison: *p* = 1).

There was no brood size‐specific (brood size category × immune challenge category, *p* = 0.76) or overall effect of the immune challenge (*p* = 0.97) or brood size category (*p* = 0.20) on mean male feeding frequency from reader deployment until 8 PM. Males paired with females that were injected later in the day had lower feeding frequencies (*p* = 0.030; see Figure S5).

The effect of the immune challenge on female body mass did not differ according to brood size (brood size category × immune challenge category, *p* = 0.78) and neither brood size (*p* = 0.42) nor the immune challenge (*p* = 0.44) had an overall effect on female body mass, measured on nestling day 14 (see Figure S6). There was a seasonal decline in female body mass at nestling day 14, so that body mass decreased with 0.03 (0.06–0.01) g Julian day^−1^ (*p* = 0.011, see Supporting Information).

### 3.2 Nestling size and body mass

**FIGURE 2:**
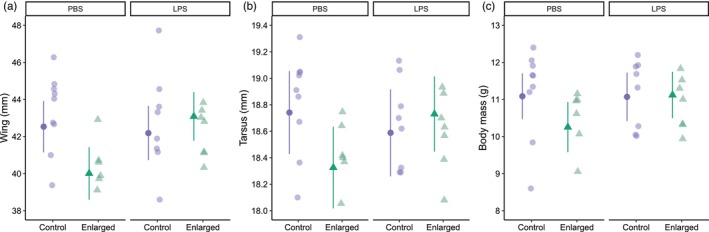
Nestling biometry of blue tits at day 14 in enlarged‐ or control broods where females where either injected with lipopolysaccharide (LPS) or phosphate‐buffered saline (PBS) when nestlings were 10 days old. Mean model estimates (±95% CI) are indicated by solid symbols and individual, raw biometrics per brood are depicted with semi‐transparent symbols.

The effect of the immune challenge on nestling wing length at day 14 was dependent on brood size (brood size category × immune challenge category, *p* = 0.018; Table 2; Figure 2a) so that nestlings from PBS‐E females had shorter wings (40.0 [38.6–41.4] mm) compared to nestlings from LPS‐E females (43.1 [41.8–44.4] mm, pairwise comparison: *p* = 0.013) and also tended to have shorter wings compared to nestlings of PBS‐C females (42.5 [41.1–43.9] mm, pairwise comparison: *p* = 0.051). Wing length did not differ between nestlings from LPS‐E females and nestlings from LPS‐C females (42.2 [40.7–43.6] mm, pairwise comparison: *p* = 0.73). There was a tendency towards a similar pattern in tarsus length (brood size category × immune challenge category, *p* = 0.074, Figure 2b) but not for body mass (*p* = 0.17; Figure 2c) and there were no main effects of neither brood size manipulation nor immune challenge category on tarsus length (Table 2; Figure 2b) or body mass (Table 2; Figure 2c). Body mass at day 6 had a strong, positive effect on all day 14 biometrics (*p* < 0.0001) and there was a seasonal decline in nestling body mass, so that body mass on day 14 decreased by 0.13 (0.21–0.04) g Julian day^−1^ (*p* = 0.007; Table 2).

## 4 DISCUSSION

The body temperature response to the immune challenge differed depending on brood size: Females challenged with a mimicked bacterial infection raising a normal‐sized brood (LPS‐C) mounted a fever that subsided, whereas females challenged with the same mimicked bacterial infection, but raising an experimentally enlarged brood (LPS‐E), initially responded with hypothermia but gradually converged with LPS‐C females approximately 6–8 h after injection. Saline solution injected females with different brood sizes did not differ in their body temperature response over time. The hypothermic response in LPS‐E females parallels the commonly observed, day‐time body temperature response in small songbirds (Burness et al., 2010; King & Swanson, 2013; Owen‐Ashley et al., 2006; Sköld‐Chiriac et al., 2015) while the hyperthermic response in LPS‐C females is more commonly observed during night‐time (but see Adelman, Bentley, et al., 2010; Adelman, Córdoba‐Córdoba, et al., 2010; Tapper et al., 2022 for hyperthermic, day‐time responses). However, in its ecological context, the relative cost of an immune response may differ (Adelman & Martin, 2009; Lee, 2006) and fever and hypothermia could both be adaptive strategies, used depending on context. The benefits of fever would likely outweigh the costs when energy is not limited, while hypothermia, potentially resulting in higher tolerance of bacterial diseases (Ganeshan et al., 2019), may be beneficial in situations where the individual is energetically constrained (Amaral‐Silva et al., 2022; Romanovsky & Székely, 1998). We suggest that this could explain the observed pattern in body temperature responses over time where LPS‐C females, tending normal‐sized broods, initially exhibited a febrile response while LPS‐E females, tending enlarged broods and therefore being under higher energetic constraint, instead responded with hypothermia. Interestingly, this difference in body temperature response to the immune challenge was likely not driven by differences in work‐induced thermogenesis, as feeding frequency did not differ between females of different brood size and immune challenge categories. The differential response to the immune challenge was further supported by female haptoglobin concentrations 4 days after the experimental injections. LPS‐C females, that initially responded with fever, had higher levels of haptoglobin compared to LPS‐E females that responded with hypothermia. Thus, our interpretation is that LPS‐E females mounted a lesser immune response, manifested by both initial hypothermia and lower circulating haptoglobin concentrations.

The brood size manipulation produced the expected pattern on nestling development in the PBS‐group (where nestlings in enlarged broods tended to have shorter wings) but not in the LPS‐group where nestlings were similarly sized, regardless of brood size and where LPS‐E nestlings had longer wings compared to PBS‐E nestlings. Wing length is an important predictor for post‐fledging survival in several bird species (Aastrup et al., 2023; Gerritsma et al., 2022; Jones et al., 2017; Jones & Ward, 2020; Martin, 2014, 2015; Morrison et al., 2009). Thus, our results suggest that LPS‐E females boosted reproductive investment and structural growth of nestlings, thereby adding to the value of their current reproduction by increasing post‐fledging survival probability. However, this increased investment was not manifested in increased feeding frequency on the day of injection which indicate that LPS‐E females increased their reproductive efforts over the days after injection and that the cumulative effect showed in nestling wing length at nestling day 14. Individuals that perceive their survival prospects to the next breeding event as low (i.e. a decreased residual reproductive value) would theoretically benefit from investing more in their current reproductive event (sensu Clutton‐Brock, 1984). Such examples of terminal investment due to a perceived sickness have been shown in several other species (Bonneaud et al., 2004; Bowers et al., 2012, 2015; Hanssen, 2006; Pärt et al., 1992; Sköld‐Chiriac et al., 2019; Velando et al., 2006). Here, LPS‐C females responded with fever and increased haptoglobin levels (compared to LPS‐E females) but raised nestlings that did not differ in size from those raised by PBS‐C females. Thus, the immune challenge alone was not enough to induce ‘terminal investment’. Such an effect was only detectable when females were simultaneously faced with two challenges: increased workload and an immune challenge.

## 5 CONCLUSIONS

We have shown that physiological responses to an endotoxin challenge, measured as both body temperature and circulating haptoglobin, may be modulated by current parental effort. Notably, females that were ‘doubly’ challenged (i.e. both by tending an enlarged brood and mounting an immune response) increased reproductive investment so that wing length, an important predictor of post‐fledging survival, of nestlings was comparable to that of nestlings in normal sized broods. Our results indicate that workload can shift the trade‐off between self‐maintenance and current reproduction when parents face a pathogenic challenge. The direction and magnitude of such a shift would likely depend on the overall life history and general life expectancy of the species, and future work could usefully expand this framework into more long‐lived species of both birds and other taxa.

## AUTHOR CONTRIBUTIONS

*Conceptualization*: Fredrik Andreasson, Arne Hegemann, Andreas Nord and Jan‐Åke Nilsson. *Methodology*: Fredrik Andreasson, Arne Hegemann, Andreas Nord and Jan‐Åke Nilsson. *Formal analysis*: Fredrik Andreasson. *Investigation*: Fredrik Andreasson, Arne Hegemann, Andreas Nord and Jan‐Åke Nilsson. *Data curation*: Fredrik Andreasson. *Writing—original draft*: Fredrik Andreasson. *Writing—review and editing*: Fredrik Andreasson, Arne Hegemann, Andreas Nord and Jan‐Åke Nilsson. *Visualization*: Fredrik Andreasson. *Supervision*: Andreas Nord and Jan‐Åke Nilsson. *Project administration*: Fredrik Andreasson and Jan‐Åke Nilsson. *Funding acquisition*: Fredrik Andreasson, Andreas Nord and Jan‐Åke Nilsson.

## CONFLICT OF INTEREST STATEMENT

The authors declare no conflicts of interest.

## Supporting information


**Table S1:** Estimates, test statistics and *p*‐values for models on biometry of nestlings in enlarged broods. Nestlings were either moved (category: Moved) in the brood size manipulation or remained in their original nest‐box (category: Stayed).
**Table S2:** Nest‐box specific information on key variables and inclusion/exclusion criteria (1 = yes, 0 = no).
**Figure S1:** Mean nestling (a) body mass, (b) wing length and (c) tarsus length of nestlings in enlarged broods that were either moved (category: Moved) in the brood size manipulation or remained in their original nest‐box (category: Stayed).
**Figure S2:** Subcutaneous body temperature (*T*_s_) for all female blue tits (panel number = nest‐box number for each female) included in analyses of *T*_s_ and feeding frequency on the day of reader deployment and injection.
**Figure S3:** Latency (i.e. time passed from reader deployment and injection until female blue tits returned to feed nestlings) was analyzed using a linear model with latency as the dependent variable, brood size‐ and immune challenge category (and the interaction between the two) as fixed factors and hatching date (Julian day) and time of injection as covariates.
**Figure S4:** Correlations between measurements of wing‐ and tarsus length of 58 adult blue tits for the two observers.
**Figure S5:** Feeding frequency in blue tit males with enlarged‐ or control brood sizes, from reader deployment to 8 PM after their partner, the female, was injected with LPS or PBS.
**Figure S6:** Body mass of female blue tits on nestling day 14.
**Figure S7:** Distribution of time between recordings for seven blue tit females that roosted in the nest‐box during the night after injection.

## Data Availability

Data and code available from figshare https://figshare.com/s/6ebadf371e8c28751e3f (Andreasson et al., 2025).
