# Supplementary material for: Current reproductive effort shapes the response to infection in a passerine bird
Source: J Anim Ecol. 2025 Jun 27;94(9):1770–83. doi: 10.1111/1365-2656.70086 (PMC12424287; doi:10.1111/1365-2656.70086)
Supplement: Supplementary file 1 — Table S1: Estimates, test statistics and p‐values for models on biometry of nestlings in enlarged broods. Nestlings were either moved (category: Moved) in the brood size manipulation or remained in their original nest‐box (category: Stayed). Table S2: Nest‐box specific information on key variables and inclusion/exclusion criteria (1 = yes, 0 = no). Figure S1: Mean nestling (a) body mass, (b) wing length and (c) tarsus length of nestlings in enlarged broods that were either moved (category: Moved) in the brood size manipulation or remained in their original nest‐box (category: Stayed). Figure S2: Subcutaneous body temperature (T s) for all female blue tits (panel number = nest‐box number for each female) included in analyses of T s and feeding frequency on the day of reader deployment and injection. Figure S3: Latency (i.e. time passed from reader deployment and injection until female blue tits returned to feed nestlings) was analyzed using a linear model with latency as the dependent variable, brood size‐ and immune challenge category (and the interaction between the two) as fixed factors and hatching date (Julian day) and time of injection as covariates. Figure S4: Correlations between measurements of wing‐ and tarsus length of 58 adult blue tits for the two observers. Figure S5: Feeding frequency in blue tit males with enlarged‐ or control brood sizes, from reader deployment to 8 PM after their partner, the female, was injected with LPS or PBS. Figure S6: Body mass of female blue tits on nestling day 14. Figure S7: Distribution of time between recordings for seven blue tit females that roosted in the nest‐box during the night after injection. [file JANE-94-1770-s001.docx]

**Fig S1.** Mean nestling (a) body mass, (b) wing length and (c) tarsus length of nestlings in enlarged broods that were either moved (category: Moved) in the brood size manipulation or remained in their original nest-box (category: Stayed). Each pair of dots connected with a line represents mean nestling biometry for nestlings in a nest-box. Mean model estimates (± 95% CI) are indicated by black symbols, raw data points are depicted with grey symbols. For all biometric measurements, we tested whether there was a difference between moved and non-moved nestlings using an individual level, mixed model with the biometric measure as dependent variable, moved or stayed and immune-challenge category as categorical, independent factors (together with the interaction between these two factors), body mass at day 6 and hatching date (Julian day) as covariates and nest-box ID as a random factor (see Table S1). The only significant difference in nestling biometry related to moving of nestlings was that, in LPS-E broods, nestlings that were transferred had a lower body mass compared to those that were not transferred (pairwise comparison: p < 0.001).

**
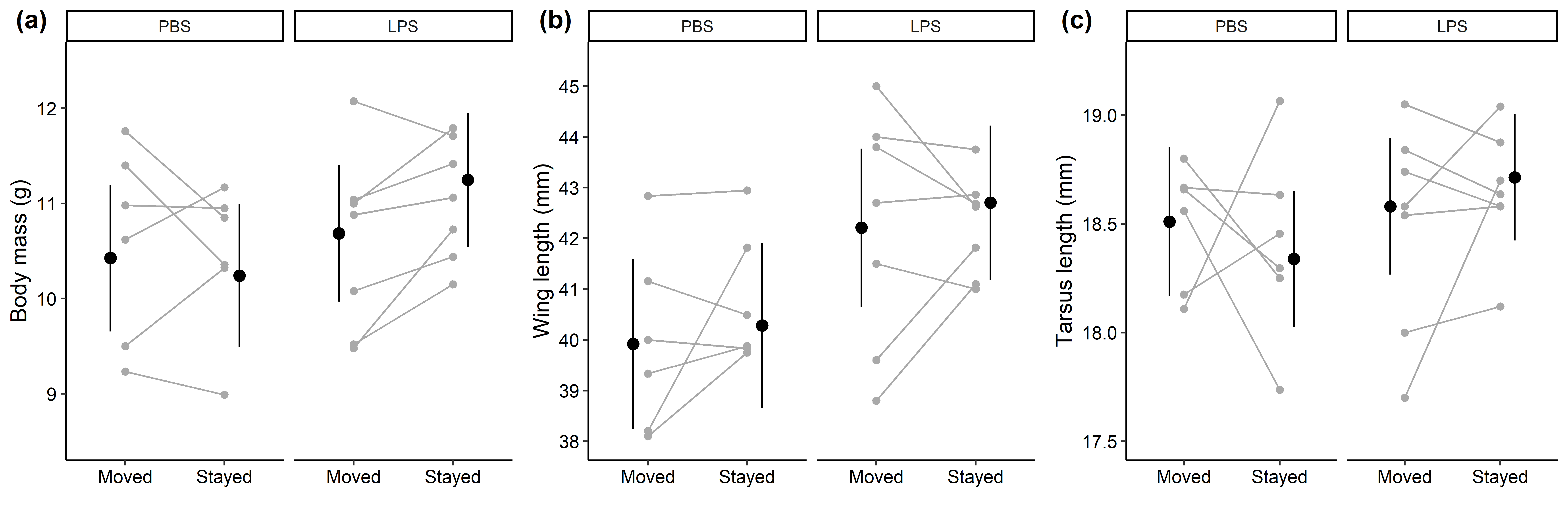
**

**Table S1.** Estimates, test statistics and p-values for models on biometry of nestlings in enlarged broods. Nestlings were either moved (category: Moved) in the brood size manipulation or remained in their original nest-box (category: Stayed). Significance values where p < 0.05 are given in bold font, and values where 0.1 > p > 0.05 are given in italics. Estimates are estimated marginal means and slopes with their 95% confidence intervals (CI).

| Variable | | Estimate (95% CI) | df | F/χ^2^ | p |
| --- | --- | --- | --- | --- | --- |
| **Nestling body mass day 14 (g)** | |  |  |  |  |
| Immune challenge | |  | 1, 10.2 | 1.8 | 0.21 |
| Moved or stayed | |  | 1, 160.8 | 3.3 | *0.073* |
| Immune challenge × Moved or stayed | |  | 1, 161.2 | 12.4 | **<0.001** |
|  | PBS – Moved | 10.43 (9.66 – 11.20) |  |  |  |
|  | PBS – Stayed | 10.24 (9.49 – 10.99) |  |  |  |
|  | LPS – Moved | 10.69 (9.97 – 11.40) |  |  |  |
|  | LPS – Stayed | 11.25 (10.55 – 11.95) |  |  |  |
| Hatching (Julian) date | | -0.09 (-0.24 – 0.07) | 1, 9.9 | 1.6 | 0.24 |
| Body mass day 6 | | 0.68 (0.57 – 0.80) | 1, 162.3 | 135.3 | **<0.0001** |
| Nest-box (random) | |  | 1 | 109.4 | **<0.0001** |
| **Nestling wing length day 14 (mm)** | |  |  |  |  |
| Immune challenge | |  | 1, 10.0 | 5.5 | **0.041** |
| Moved or stayed | |  | 1, 160.8 | 2.8 | *0.094* |
| Immune challenge × Moved or stayed | |  | 1, 161.3 | 0.1 | 0.80 |
|  | PBS – Moved | 39.9 (38.2 – 41.6) |  |  |  |
|  | PBS – Stayed | 40.3 (38.7 – 41.9) |  |  |  |
|  | LPS – Moved | 42.2 (40.7 – 43.8) |  |  |  |
|  | LPS – Stayed | 42.7 (41.2 – 44.2) |  |  |  |
| Hatching (Julian) date | | 0.02 (-0.32 – 0.35) | 1, 9.7 | 0.0 | 0.92 |
| Body mass day 6 | | 2.35 (2.07 – 2.63) | 1, 162.7 | 273.0 | **<0.0001** |
| Nest-box (random) | |  | 1 | 76.8 | **<0.0001** |
| **Nestling tarsus length day 14 (mm)** | |  |  |  |  |
| Immune challenge | |  | 1, 10.6 | 1.3 | 0.28 |
| Moved or stayed | |  | 1, 162.6 | 0.1 | 0.81 |
| Immune challenge × Moved or stayed | |  | 1, 163.7 | 3.5 | *0.064* |
|  | PBS – Moved | 18.51 (18.17 – 18.85) |  |  |  |
|  | PBS – Stayed | 18.34 (18.03 – 18.65) |  |  |  |
|  | LPS – Moved | 18.58 (18.27 – 18.89) |  |  |  |
|  | LPS – Stayed | 18.71 (18.42 – 19.01) |  |  |  |
| Hatching (Julian) date | | 0.004 (-0.061 – 0.068) | 1, 9.7 | 0.0 | 0.90 |
| Body mass day 6 | | 0.38 (0.29 – 0.47) | 1, 166.8 | 72.0 | **<0.0001** |
| Nest-box (random) | |  | 1 | 28.5 | **<0.0001** |

**Fig S2.** Subcutaneous body temperature (*T*_s_) for all female blue tits (panel number = nest-box number for each female) included in analyses of *T*_s_ and feeding frequency on the day of reader deployment and injection. Blue line indicates injection time and red line indicates 8pm, after which time recordings were excluded from analyses to avoid including drops in *T*_s_ and feeding frequency during the period before going to roost, which would introduce variation unrelated to the experimental effect. Females with enlarged broods have green panel headings and females with control broods have purple panel headings.


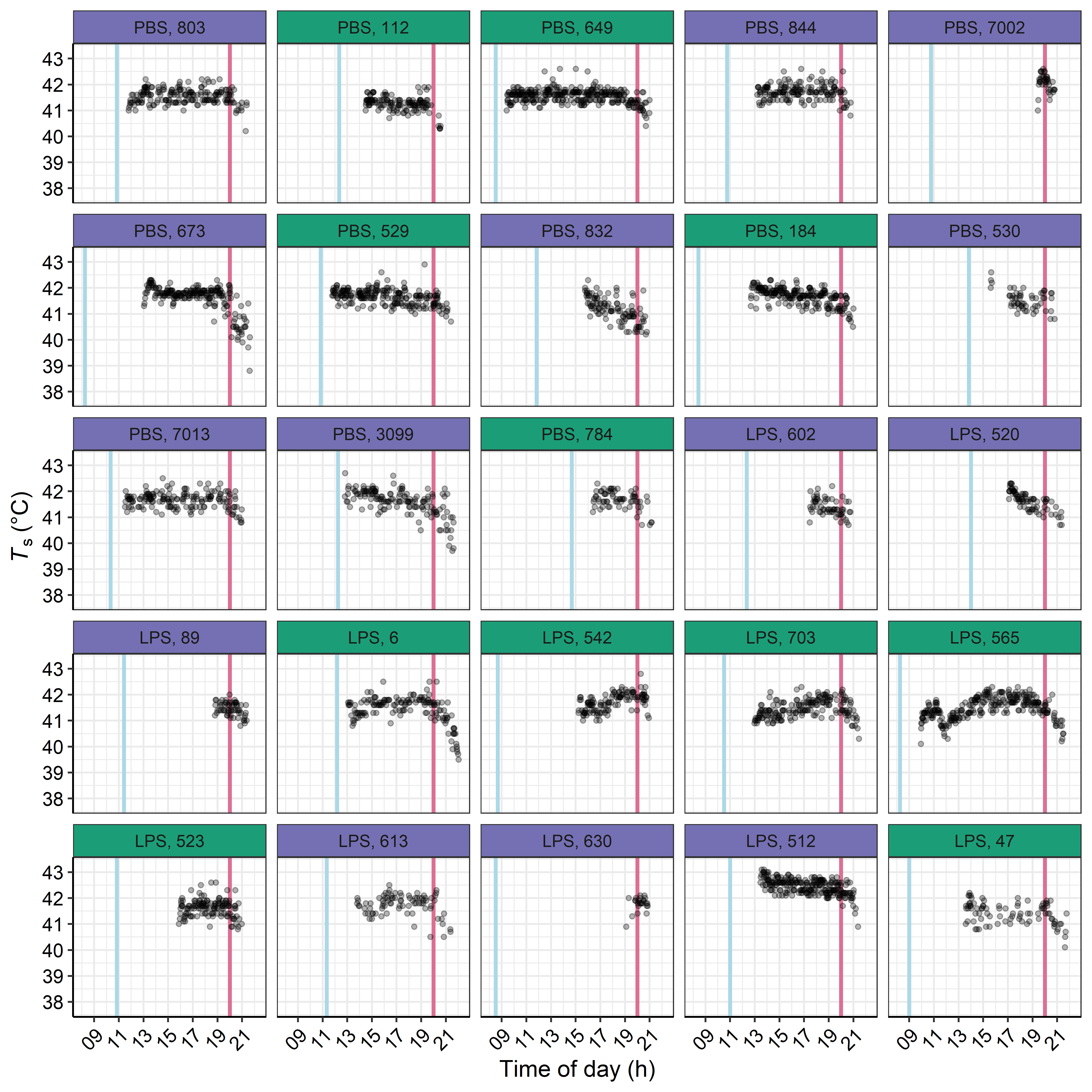


**Table S2.** Nest-box specific information on key variables and inclusion/exclusion criteria (1 = yes, 0 = no).

| Nestbox | Brood size | Immune  challenge | Brood size  pre manipulation | Brood size  post  manipulation | Brood size  day 14 | Female on  reader day 10 | Female on  reader | Female  feeding  day 14 | Male  feeding  day 14 | Included in  day 14  analyses | Note |
| --- | --- | --- | --- | --- | --- | --- | --- | --- | --- | --- | --- |
| 803 | Control | PBS | 9 | 9 | 9 | 1 | 1 | 1 | 1 | 1 |  |
| 830 | Enlarged | PBS | 9 | 14 |  | 0 | 0 | 0 | 0 | 0 |  |
| 596 | Control | LPS | 9 | 9 | 9 | 0 | 1 | 1 | 1 | 1 |  |
| 112 | Enlarged | PBS | 11 | 16 | 15 | 1 | 1 | 1 | 1 | 1 |  |
| 602 | Control | LPS | 10 | 10 | 10 | 1 | 1 | 1 | 1 | 1 |  |
| 649 | Enlarged | PBS | 10 | 15 | 15 | 1 | 1 | 1 | 1 | 1 |  |
| 844 | Control | PBS | 9 | 9 | 9 | 1 | 1 | 1 | 1 | 1 |  |
| 7004 | Enlarged | LPS | 11 | 16 | 16 | 0 | 0 | 1 | 1 | 1 |  |
| 520 | Control | LPS | 10 | 10 | 10 | 1 | 1 | 0 | 1 | 0 |  |
| 89 | Control | LPS | 9 | 9 | 9 | 1 | 1 | 1 | 1 | 1 |  |
| 732 | Enlarged | PBS | 9 | 14 | 13 | 0 | 1 | 1 | 1 | 1 |  |
| 7002 | Control | PBS | 9 | 9 | 9 | 1 | 1 | 1 | 1 | 1 |  |
| 6 | Enlarged | LPS | 11 | 16 | 15 | 1 | 1 | 1 | 0 | 0 |  |
| 7017 | Control | LPS | 12 | 12 | 12 | 0 | 1 | 1 | 1 | 1 |  |
| 199 | Enlarged | LPS | 10 | 15 | 15 | 0 | 1 | 1 | 1 | 1 |  |
| 542 | Enlarged | LPS | 11 | 16 | 12 | 1 | 1 | 1 | 1 | 1 |  |
| 703 | Enlarged | LPS | 10 | 15 | 15 | 1 | 1 | 1 | 1 | 1 |  |
| 629 | Control | PBS | 12 | 12 |  | 0 | 0 | 0 | 0 | 0 | Predation |
| 825 | Enlarged | PBS | 10 | 15 | 14 | 0 | 1 | 1 | 1 | 1 |  |
| 673 | Control | PBS | 11 | 11 | 11 | 1 | 1 | 1 | 1 | 1 |  |
| 574 | Enlarged | LPS | 11 | 16 |  | 0 | 0 | 0 | 0 | 0 |  |
| 565 | Enlarged | LPS | 11 | 16 | 16 | 1 | 1 | 1 | 1 | 1 |  |
| 529 | Enlarged | PBS | 10 | 15 | 14 | 1 | 1 | 1 | 1 | 1 |  |
| 832 | Control | PBS | 11 | 11 | 11 | 1 | 1 | 1 | 1 | 1 |  |
| 523 | Enlarged | LPS | 12 | 17 | 17 | 1 | 1 | 1 | 1 | 1 |  |
| 184 | Enlarged | PBS | 10 | 15 | 12 | 1 | 1 | 0 | 1 | 0 |  |
| 613 | Control | LPS | 11 | 11 | 11 | 1 | 1 | 1 | 1 | 1 |  |
| 530 | Control | PBS | 9 | 9 | 9 | 1 | 1 | 1 | 1 | 1 |  |
| 817 | Enlarged | LPS | 11 | 16 | 7 | 0 | 0 | 0 | 1 | 0 |  |
| 7013 | Control | PBS | 10 | 10 | 10 | 1 | 1 | 1 | 1 | 1 |  |
| 630 | Control | LPS | 11 | 11 | 11 | 1 | 1 | 1 | 1 | 1 |  |
| 3099 | Control | PBS | 10 | 10 | 7 | 1 | 1 | 1 | 1 | 1 |  |
| 3009 | Enlarged | LPS | 11 | 16 |  | 0 | 0 | 0 | 0 | 0 |  |
| 784 | Enlarged | PBS | 9 | 14 | 11 | 1 | 1 | 1 | 1 | 1 |  |
| 512 | Control | LPS | 13 | 13 | 12 | 1 | 1 | 1 | 1 | 1 |  |
| 3145 | Control | LPS | 11 | 11 |  | 0 | 0 | 0 | 0 | 0 |  |
| 3146 | Control | PBS | 10 | 10 | 6 | 0 | 1 | 1 | 1 | 1 |  |
| 1028 | Control | LPS | 11 | 11 | 10 | 0 | 1 | 1 | 1 | 1 |  |
| 47 | Enlarged | LPS | 10 | 15 | 15 | 1 | 1 | 1 | 1 | 1 |  |

**Fig S3.** Latency (i.e. time passed from reader deployment and injection until female blue tits returned to feed nestlings) was analyzed using a linear model with latency as the dependent variable, brood size- and immune challenge category (and the interaction between the two) as fixed factors and hatching date (Julian day) and time of injection as covariates. There was no brood size dependent effect of treatment on latency (interaction: brood size category × immune challenge category, p = 0.51) and neither brood size (p = 0.093) nor immune challenge category (p = 0.12) had a main effect on latency. Mean model estimates (± 95% CI) are indicated by solid symbols and individual, raw data points are depicted with semi-transparent symbols.

**
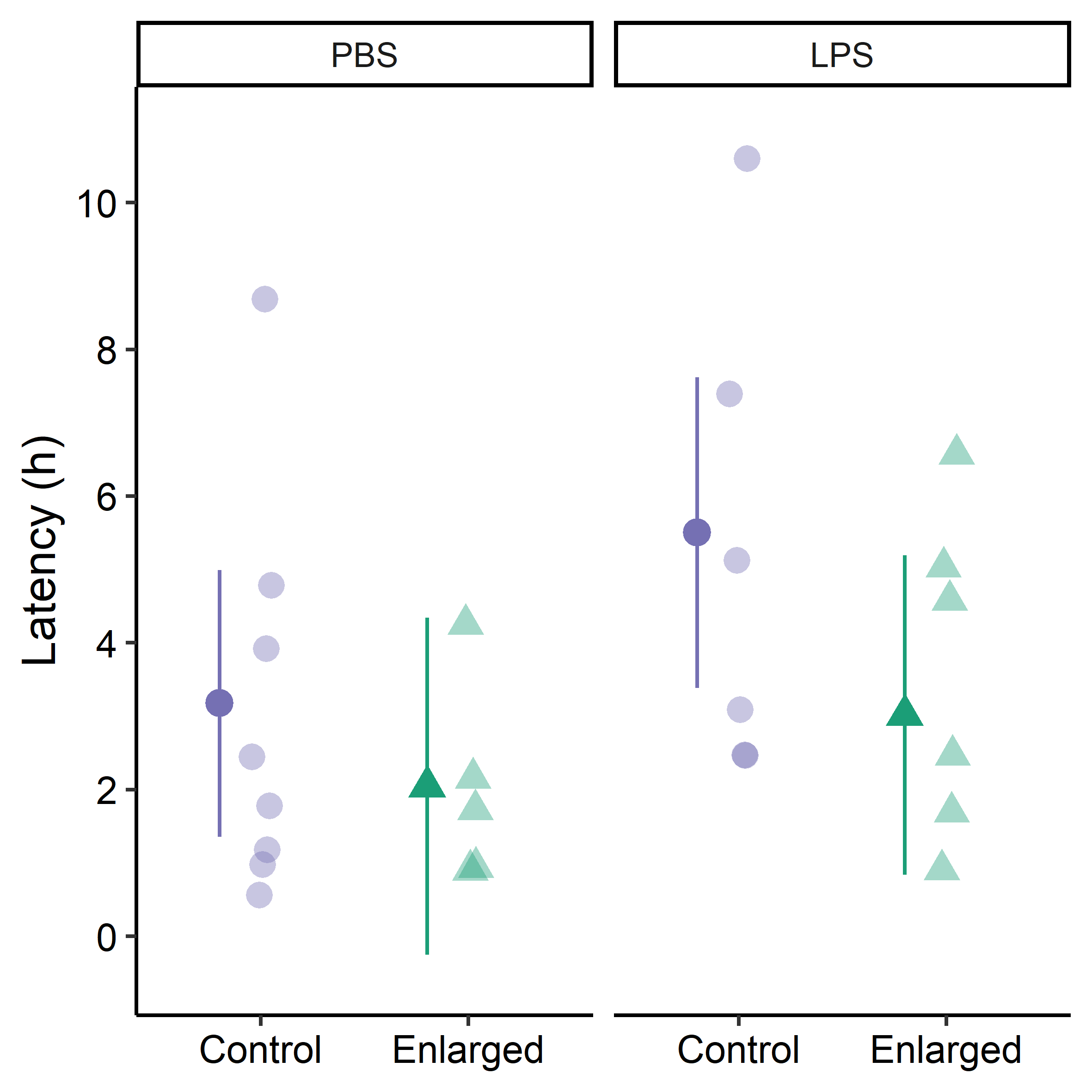
**

| Variable | | Estimate (95% CI) | df | F | p |
| --- | --- | --- | --- | --- | --- |
| **Latency (h)** | |  |  |  |  |
| Immune challenge | |  | 1, 19 | 2.7 | 0.12 |
| Brood size | |  | 1, 19 | 3.1 | *0.093* |
| Immune challenge × Brood size | |  | 1, 19 | 0.4 | 0.51 |
|  | PBS – Control | 3.2 (1.4 – 5.0) |  |  |  |
|  | PBS – Enlarged | 2.0 (-0.3 – 4.3) |  |  |  |
|  | LPS – Control | 5.5 (3.4 – 7.6) |  |  |  |
|  | LPS – Enlarged | 3.0 (0.8 – 5.2) |  |  |  |
| Hatching (Julian) date | | -0.001 (-0.291 – 0.289) | 1, 19 | 0.0 | 0.99 |
| Time of injection | | -0.55 (-1.15 – 0.04) | 1, 19 | 3.8 | *0.068* |

**Fig S4.** Correlations between measurements of wing- and tarsus length of 58 adult blue tits for the two observers. Equations were used to transform measurements made by FA to the same scale as those made by AN. Dashed line represents y = x.

**
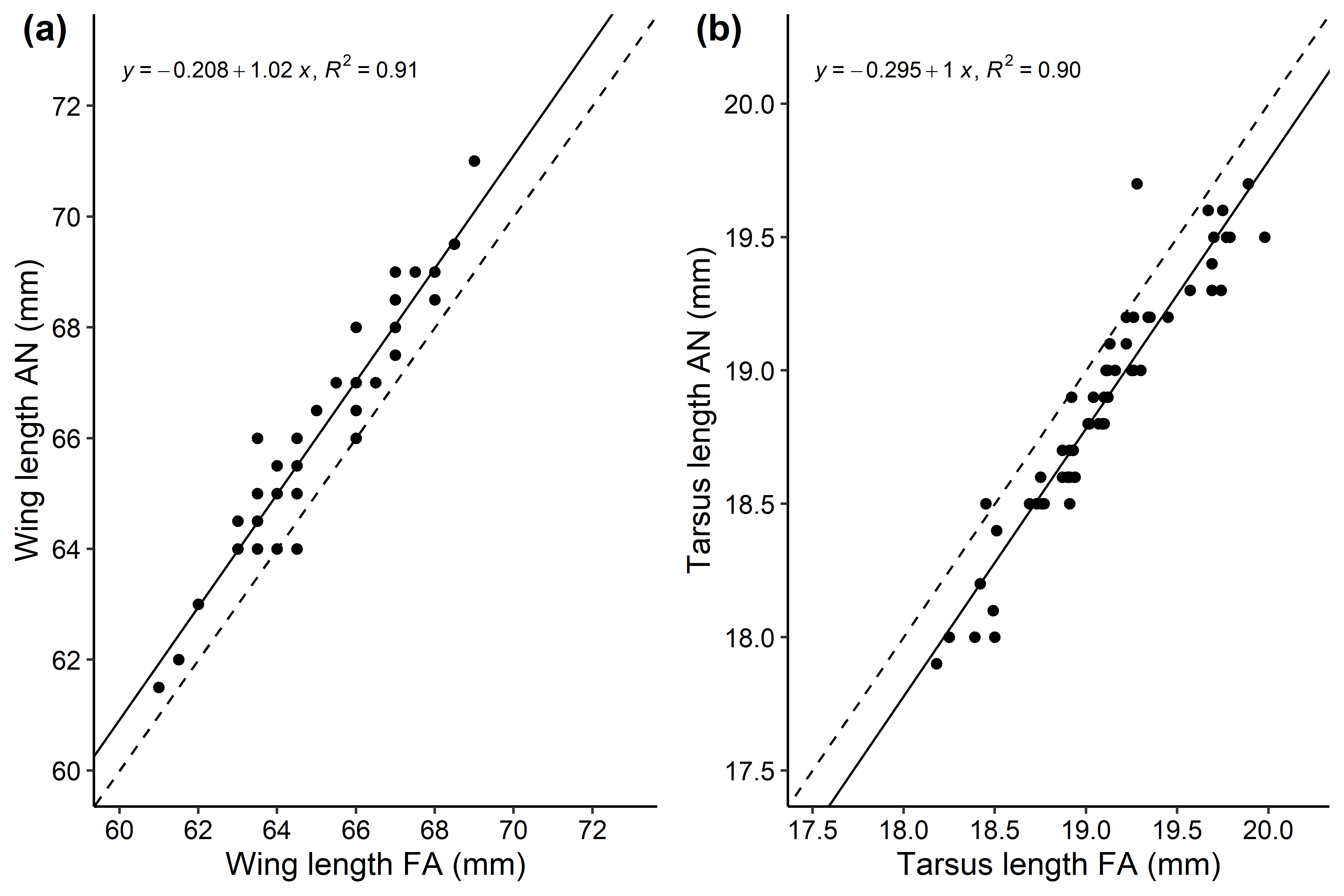
**

**Fig S5.** Feeding frequency in blue tit males with enlarged- or control brood sizes, from reader deployment to 8 pm after their partner, the female, was injected with LPS or PBS. Mean model estimates (± 95% CI) are indicated by solid symbols and individual, raw data points are depicted with semi-transparent symbols.

**
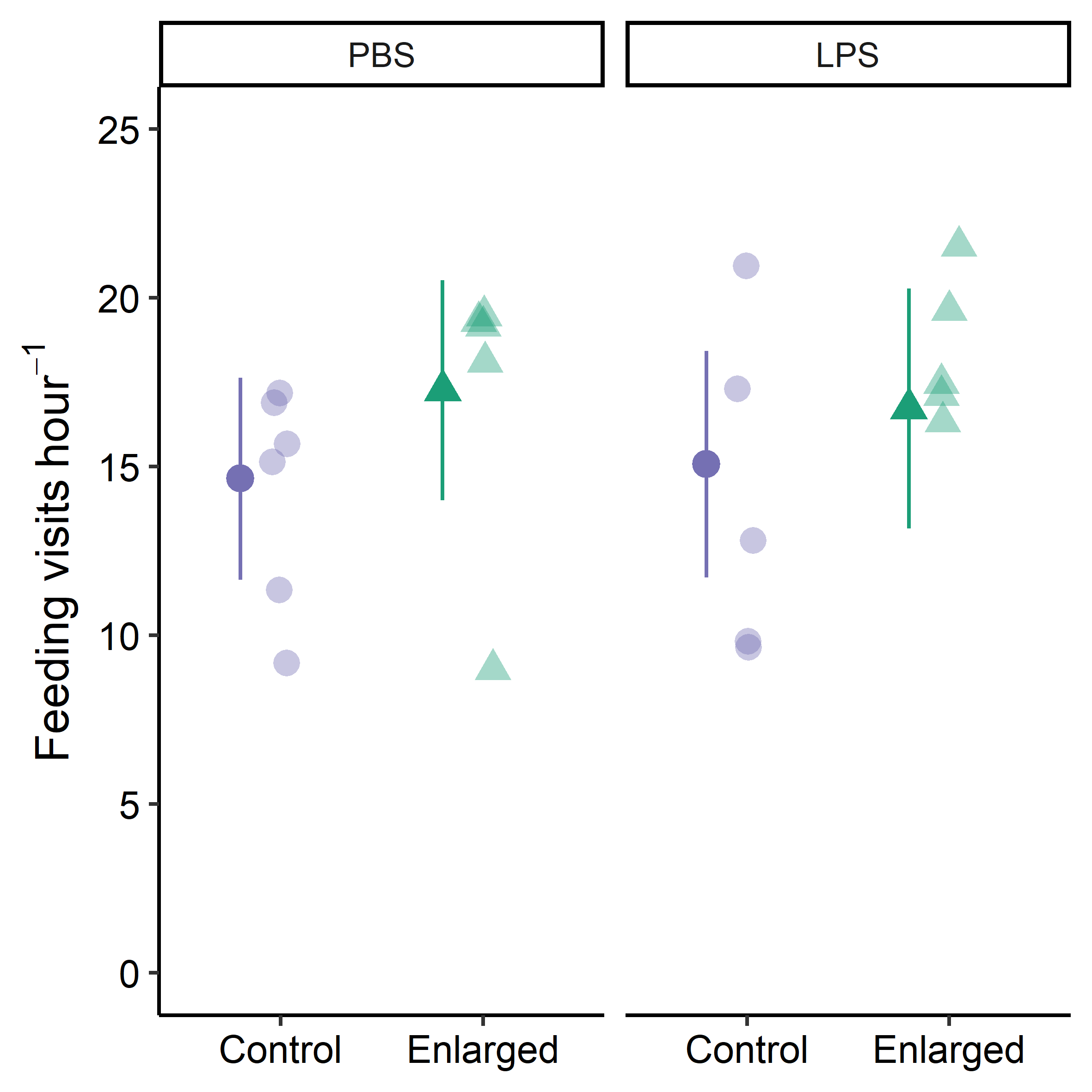
**

| Variable | | Estimate (95% CI) | df | F | p |
| --- | --- | --- | --- | --- | --- |
| **Male feeding frequency (visits h^-1^)** | |  |  |  |  |
| Immune challenge | |  | 1, 15 | 0.0 | 0.97 |
| Brood size | |  | 1, 15 | 1.8 | 0.20 |
| Immune challenge × Brood size | |  | 1, 15 | 0.1 | 0.76 |
|  | PBS – Control | 14.6 (11.7 – 17.6) |  |  |  |
|  | PBS – Enlarged | 17.3 (14.0 – 20.5) |  |  |  |
|  | LPS – Control | 15.1 (11.7 – 18.4) |  |  |  |
|  | LPS – Enlarged | 16.7 (13.2 – 20.3) |  |  |  |
| Hatching (Julian) date | | 0.23 (-0.26 – 0.72) | 1, 15 | 1.0 | 0.34 |
| Time of injection | | -1.0 (-1.9 – -0.1) | 1, 15 | 5.7 | **0.030** |

**Fig S6.** Body mass of female blue tits on nestling day 14. Panels show females that were injected with LPS or PBS on nestling day 10, separated on brood size. Mean model estimates (± 95% CI) are indicated by solid symbols and individual, raw data points are depicted with semi-transparent symbols.

**
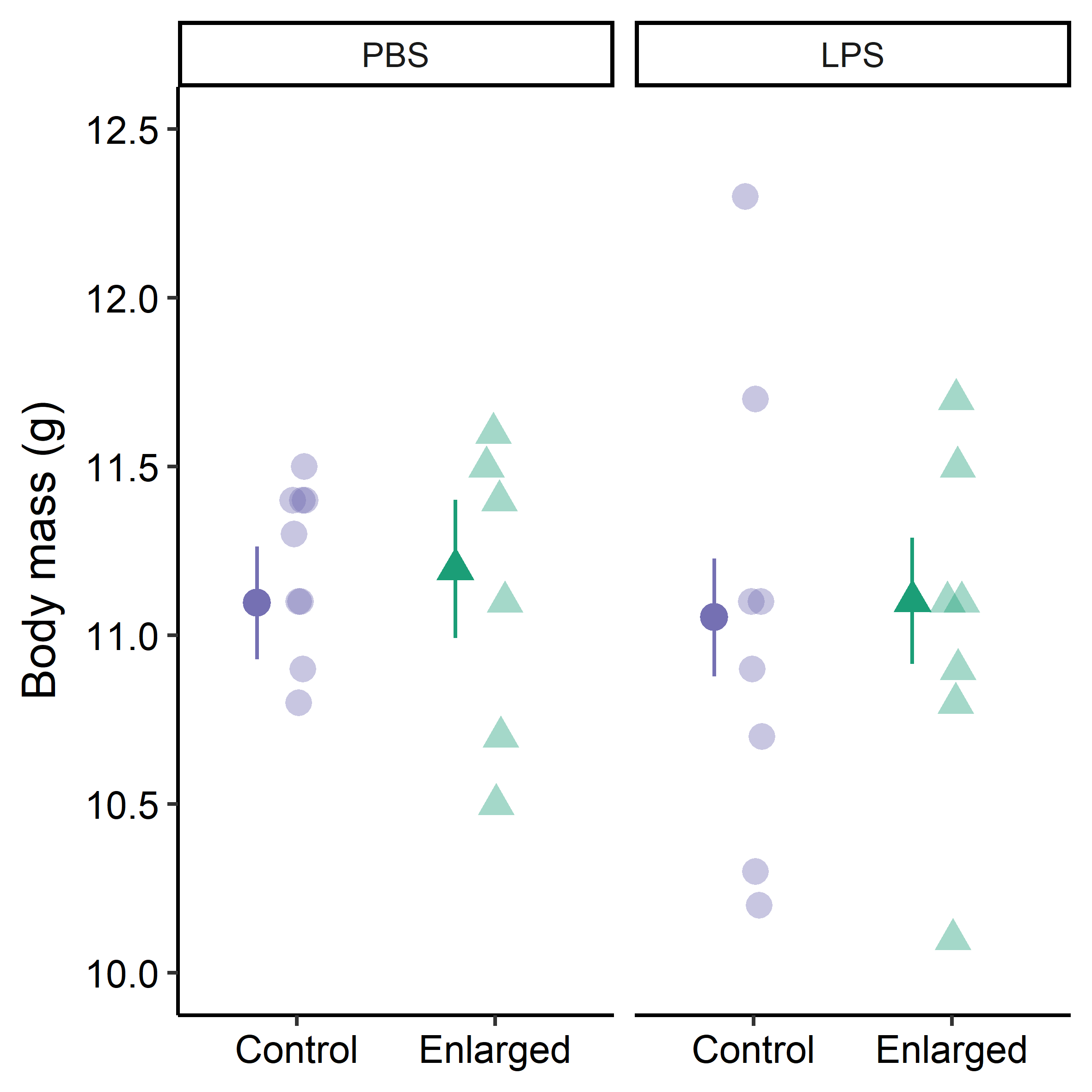
**

| Variable | | Estimate (95% CI) | df | F | p |
| --- | --- | --- | --- | --- | --- |
| **Female body mass day 14 (g)** | |  |  |  |  |
| Immune challenge | |  | 1, 24 | 0.6 | 0.44 |
| Brood size | |  | 1, 24 | 0.7 | 0.42 |
| Immune challenge × Brood size | |  | 1, 24 | 0.1 | 0.78 |
|  | PBS – Control | 11.10 (10.93 – 11.26) |  |  |  |
|  | PBS – Enlarged | 11.20 (10.99 – 11.40) |  |  |  |
|  | LPS – Control | 11.05 (10.88 – 11.23) |  |  |  |
|  | LPS – Enlarged | 11.10 (10.92 – 11.29) |  |  |  |
| Hatching (Julian) date | | -0.03 (-0.06 – -0.01) | 1, 24 | 7.7 | **0.011** |
| Body mass day 10 | | 1.13 (0.87 – 1.38) | 1, 24 | 85.9 | **<0.0001** |

**Description of filtering of feeding frequency recordings**

A feeding visit and the corresponding temperature measurement (for females *T*_s_ and for males temperature measured by the leg-mounted PIT-tag) was recorded each time a bird visited and entered the nest-box. The antenna had a reading range of approximately ≤ 20 cm. Readers were set to record individual PIT-tags, but with a refractory period of 60 s. Thus, a new recording could only be made 60 s after the initial recording. However, this could potentially create false double readings (see Iserbyt et al. 2018 and Andreasson et al. 2020 for a more detailed discussion). Therefore, we adopted a similar approach as in Andreasson et al. (2020) to filter out these potential double readings. Seven females roosted in the nest-box on the night after injection and by using data from these females (that were continuously recorded by the antenna with a refractory period of 60 s) we could conclude that 90.3% of all recordings (where we know that the female was within reading range) were registered 60-70 s after the previous recording and 86.4% of all recordings were registered 60-62 s after the previous recording (Fig S6). Therefore, we chose to define the 60-62 s interval as possible double readings and filtered the data accordingly (see below).

For males, we listed all readings that were within 60-62 s of one another (potential double readings) and excluded those where the temperature reading from the leg-mounted PIT-tag exceeded 30 °C (indicating that the bird had remained in the nest-box during the 60 s refractory period).

For females, we also listed all readings that were within 60-62 s of one another and if there were more than two such readings in succession all readings after the first one were removed. I.e. if a female was recorded four times, where each reading was within 60-62 seconds of the preceding reading all but the first reading were removed. For females that roosted together with the nestlings in the nest-box, night-time recordings were manually removed. Start of night was defined as when the female had been in the nest-box for > 5 min, and these recordings (n = 5) were also removed.

**Fig S7.** Distribution of time between recordings for seven blue tit females that roosted in the nest-box during the night after injection. In total, 2469 readings were recorded. **a)** Shows all data and **b)** shows only recordings that were registered 60-70 s after the previous recording.

**
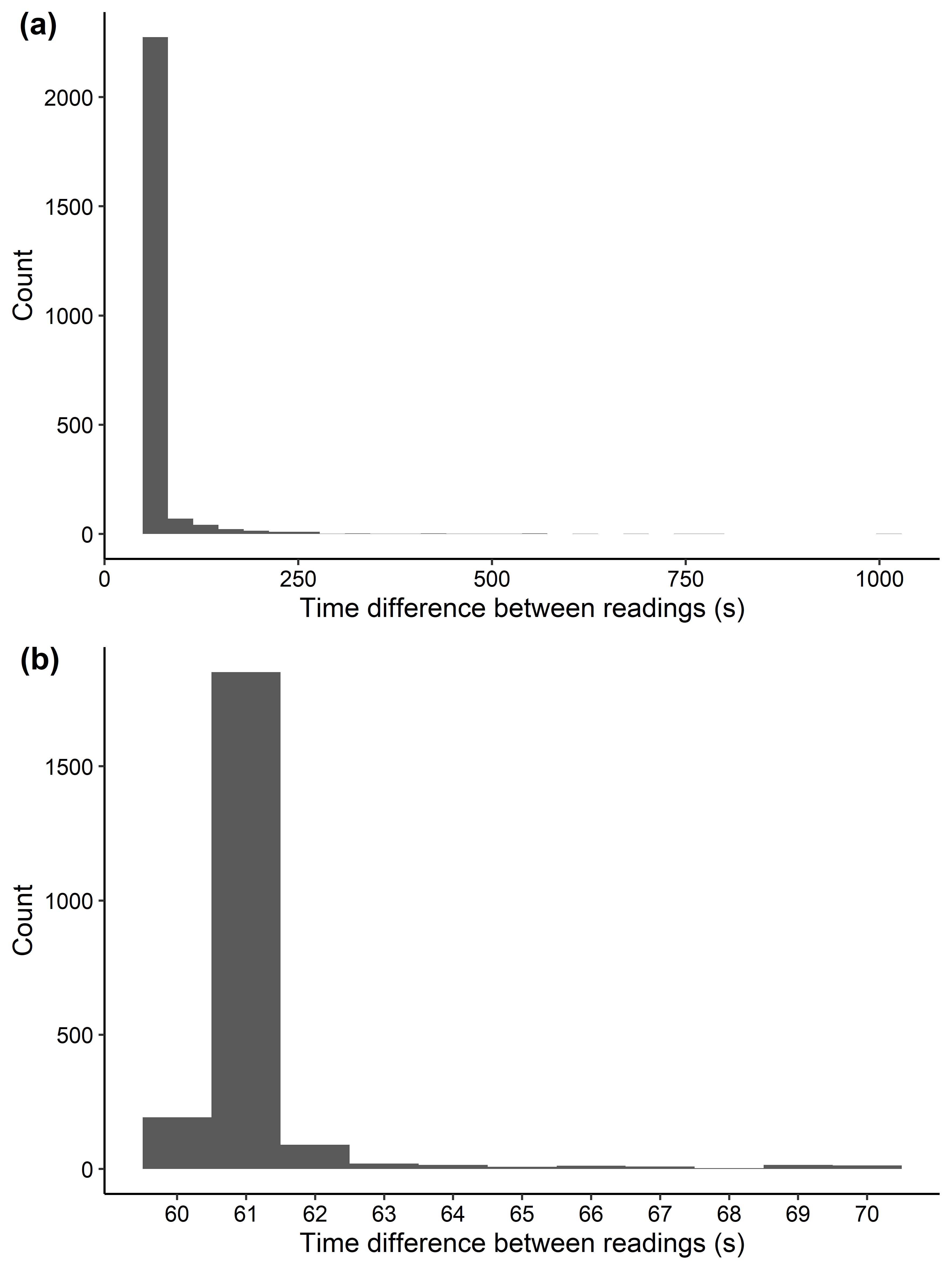
**

**References**

Andreasson F, Hegemann A, Nord A, Nilsson JÅ (2020). Experimental facilitation of heat loss affects work rate and innate immune function in a breeding passerine bird. J Exp Biol 223, jeb219790.

Iserbyt A, Griffioen M, Borremans B, Eens M, Müller W (2018). How to quantify animal activity from radio frequency identification (RFID) recordings. Ecol Evol 8, 10166-10174.
